# Development and pilot testing of the treatment and education approach for childhood-onset lupus (TEACH): a cognitive behavioral treatment

**DOI:** 10.1186/s12969-019-0307-8

**Published:** 2019-02-18

**Authors:** Natoshia R. Cunningham, Lauren M. Fussner, Erin Moorman, Pinar O. Avar Aydin, Hermine I. Brunner, Susmita Kashikar-Zuck

**Affiliations:** 1 University of Cincinnati College of Medicine, Cincinnati Children’s Hospital Medical Center, 3333 Burnet Ave, Cincinnati, OH 45229 USA; 2 Division of Behavioral Medicine and Clinical Psychology, MLC 7039, Cincinnati Children’s Hospital Medical Center, 3333 Burnet Ave, Cincinnati, OH 45229 USA; 3 Division of Rheumatology, Cincinnati Children’s Hospital Medical Center, 3333 Burnet Ave, Cincinnati, OH 45229 USA

**Keywords:** Childhood-onset, Lupus, Cognitive behavioral therapy (CBT), Fatigue, Depressive symptoms, Pain

## Abstract

**Background:**

To develop and test the feasibility and initial effectiveness of the Treatment and Education Approach for Childhood-onset Lupus (TEACH) protocol, a 6-session cognitive behavioral therapy (CBT) intervention for adolescents and young adults (AYA) with childhood-onset systemic lupus erythematosus (cSLE).

**Methods:**

Females with cSLE (*n* = 14; ages 13–19 years, *M* = 16.21 years) presenting to a pediatric rheumatology clinic subsequently completed the protocol, which was iteratively modified based on participant/interventionist feedback. Upon intervention completion, participants provided qualitative data on feasibility, acceptability, potential modifications, and perceived effectiveness of the program via a semi-structured interview, which was analyzed for shared themes. Participants also completed measures of fatigue, psychological distress, and pain intensity before and after the intervention. Nonparametric statistics were conducted to examine changes in outcome measures following the intervention.

**Results:**

During the study, several protocol modifications were employed to better address the unique needs of individuals with cSLE (e.g., separate content for adolescents versus young adults). Results suggest that TEACH is feasible, acceptable, and potentially effective in the management of cSLE symptoms. Following the intervention, there was a statistically significant reduction in fatigue (Z = − 2.81, *p* < .01) and depressive symptoms (Z = − 2.69, *p* < .01). Reductions in pain and anxiety symptoms were marginal.

**Conclusions:**

TEACH, a tailored CBT protocol for AYA with cSLE, is a feasible and potentially effective intervention for the management of fatigue and depressive symptoms. Future directions include testing the protocol in a larger controlled study.

## Background

Systemic lupus erythematosus (SLE) is a chronic, multisystem, inflammatory, autoimmune disease requiring frequent medical visits and a complex medical regimen [1]. Approximately 10–20% of new SLE diagnoses occur in adolescence [2], which accounts for a prevalence rate of 3.3 to 24 per 100,000 children [3]. When diagnosed prior to age 18, childhood-onset SLE (cSLE) is associated with more severe disease onset, active disease presentation, and greater use of steroid and immunosuppressive therapy [4]. A large portion of youth with cSLE also experience debilitating symptoms including fatigue (65–67%) [5, 6], depressive symptoms (30%) [6], and pain (40%) [6]. These factors complicate management of cSLE and adversely impact health-related quality of life, even when disease activity is well managed [6]. Thus, a tailored behavioral treatment approach may be useful in helping youth with cSLE manage symptoms of fatigue, psychological distress, and pain.

Cognitive behavioral interventions have been shown to be effective in the management of several pediatric chronic conditions including type 1 diabetes [7], juvenile fibromyalgia [8], and inflammatory bowel disease [9]. Following cognitive behavioral treatments for pediatric health conditions that involve pain, significant improvements in adherence, mood symptoms, and adjustment are generally observed [10]. More recent work has incorporated the use of mindfulness meditation strategies into cognitive behavioral interventions to improve pediatric health outcomes with positive results [11]. To date, only one CBT program has been tested among adolescent females with cSLE; however, there was no significant overall improvement in the CBT group as compared to control conditions [12]. This intervention was delivered almost exclusively via computerized modules and *was not tailored specifically to target the psychological difficulties common in youth with cSLE*. Psychological interventions have been developed for *adults* with SLE, and are associated with improvements in depression, anxiety, and stress [13]. Surprisingly, no studies thus far have developed treatments to address the unique developmental needs of adolescents and young adults (AYA) with cSLE.

It is critical to develop a tailored treatment specific to adolescence and early adulthood as these are vulnerable periods of development characterized by increased autonomy and changes in emotional functioning [14]. Importantly, AYA with chronic conditions may be at heightened risk for poor health-related outcomes (e.g., depressive symptoms, poor social adjustment) [15, 16]. Thus, this developmental period represents an opportune time to teach disease-specific coping strategies and foster independence in symptom management to improve psychological and health-related outcomes.

The current study aimed to develop and refine the *Treatment and Education Approach for Childhood-onset Lupus* (TEACH) protocol, a brief cognitive behavioral intervention specifically tailored for AYA with cSLE, and to assess preliminary evidence of feasibility, acceptability, and impact on symptoms of fatigue, psychological distress (i.e., depressive symptoms and anxiety symptoms), and pain. We predicted the TEACH protocol would be feasible to implement, well-accepted by participants, and lead to post-treatment improvements in fatigue, psychological distress, and pain intensity. Referral rates to psychological providers following TEACH were also explored.

## Methods

### Participants

Youth between the ages of 13 and 21 and their primary caregivers were recruited from a pediatric rheumatology clinic at a large Midwestern Children’s hospital in the United States. Eligibility criteria included English language proficiency, previous diagnosis of cSLE based on American College of Rheumatology (ACR) criteria [17], clinical levels (T-score ≥ 65; for additional details on cut-offs, see the Measures section) of fatigue and depressive symptoms, and moderate levels pain (i.e., average pain in the past week ≥4 on 0–10 Numerical Rating Scale). Inclusion based on one symptom criteria (versus all) afforded the opportunity to deliver a protocol that would broadly meet the needs of the diverse patients with cSLE. Participants with an untreated major psychiatric diagnosis (e.g., bipolar disorder, psychosis) or a documented developmental delay were not eligible to participate. A total of 34 youth were approached for the current study. Of these, 18 participants (53%) met eligibility criteria and were enrolled (94% female, *M*_age_ = 16.89, *SD*_age_ = 2.27, eight of which were ≥ 18 years). Participants were predominantly Caucasian (*n* = 11), African American (*n* = 3), and Asian American (*n* = 2). Mean duration of diagnosis at time of enrollment was 39.4 months (SD = 34.4, range 1 month – 96 months). One participant identified as more than one race and one participant identified as Hispanic/Latino. Household income ranged from less than $25,000 to greater than $100,000 with the average household income between $25,000-50,000.

### Procedure

All study procedures were approved by the Institutional Review Board. Rheumatology physicians introduced the study to patients during their routine medical visit. Interested families then met with a clinical research coordinator, who explained details of the study and gave the families opportunities to raise questions and voice concerns. Participants and their caregivers then provided informed assent/consent. Participants completed baseline (T1) self-report measures to determine study eligibility. Participants who met clinical cutoff scores on fatigue, depressive symptoms, or pain (see Measures section below) were scheduled to begin the TEACH intervention. This generally occurred within a week, but no later than 4 weeks after the initial screening. As the intervention was delivered, observations from participants and interventionists informed further refinement of the TEACH protocol. At post-treatment (T2; conducted directly after completing the intervention), participants provided feedback on TEACH (e.g., feasibility, acceptability) via a semi-structured qualitative interview and also completed self-report measures to assess initial effectiveness (see measures section below).

### Initial development of TEACH intervention

TEACH is an individual treatment protocol developed to reduce fatigue, psychological distress, and pain levels in patients with cSLE. The program included 6 weekly in-person sessions facilitated by a doctoral level psychologist. Each session lasted approximately 60 min, and caregivers were invited to attend three of the six sessions. TEACH was developed using established CBT protocols for pediatric chronic pain [8] and insomnia [18]. Given that psychological distress is common in youth with cSLE, some strategies in the pediatric chronic pain CBT protocol (e.g., calming statements, pleasant event scheduling) were refined to better address co-occurring mood symptoms. Coping skills included psychoeducation for managing physical symptoms (i.e., Gate Control Theory of Pain), sleep hygiene recommendations, relaxation training (i.e., deep breathing, guided imagery, progressive muscle relaxation), behavioral activation (i.e., pleasant activity scheduling), activity pacing, calming statements, problem solving, and maintenance planning.

Participant and therapist feedback was reviewed after every three to six participants completed the intervention to inform intervention revision. Additional program content, such as mindfulness meditation, medication adherence, and challenging automatic thoughts, was subsequently included during the iterative refinement process (see Results section below).

### Qualitative interviews

At post-treatment, a trained clinical research coordinator administered a semi-structured qualitative interview to patients to assess feasibility, acceptability, content, and format of the protocol, and to query about potential modifications. Participants answered approximately 35 questions. Sample questions include: *How is the pace of the program? How could the skills be presented better? What might get in the way of you practicing these skills on your own? Are there any other aspects of your illness that you struggle coping with that we did not address?* Qualitative interviews were recorded and subsequently transcribed for review. After all data collection was complete, three doctoral level psychologists and one post-baccalaureate clinical research coordinator reviewed qualitative interviews for convergence of themes across participants until saturation was achieved [19]. When differences arose, themes were discussed until consensus was reached. This process was informed by our previous studies [20, 21] employing qualitative thematic analysis [22] to develop/refine behavioral interventions for pediatric chronic health conditions involving pain.

### Measures

#### Demographic/background information

Participants self-reported their age, race, and ethnicity. Caregivers reported average family income. Disease duration was obtained from the participant’s medical chart.

#### Disease activity

The Systemic Lupus Erythematosus Disease Activity Index (SLEDAI) was used to assess disease activity [23]. Scores range from 0 (no activity) to 105. Participants’ rheumatologists completed the SLEDAI at the time of enrollment.

#### Fatigue

Fatigue over the previous 7 days was measured using the fixed length PROMIS fatigue self-report form [24]. Participants under 18 years completed the 10-item pediatric fatigue short form (e.g., “I got tired easily”). Items are scored on a 5-point Likert scale (0 = *never*, 4 = *almost always*) and summed to create a total score ranging from 0 to 40. On the pediatric fatigue form, a raw score ≥ 26 is consistent with a T-score ≥ 65. Participants 18 years and older completed the 8-item adult short form (e.g., “I felt exhausted”) [25, 26]. Items are scored on a 5-point Likert scale (1 = *not at all*, 5 = *very much*) and summed to create a total score ranging from 8 to 40. On the adult fatigue form, a raw score ≥ 32 is consistent with a T-score ≥ 65. Internal consistency in the current sample was very good to excellent for the PROMIS pediatric fatigue (*α* ≥ .96) and adult fatigue (*α* ≥ .87) self-report forms.

#### Psychological distress

Participants completed self-report forms to assess depressive symptoms and anxiety symptoms.

##### Depressive symptoms

The 28-item Children’s Depression Inventory 2nd Edition (CDI2) was used to assess depressive symptoms in youth under 18 years of age [27]. For each item, participants select one of three statements that best describe them over the previous two weeks (e.g., “I like myself,” “I do not like myself,” “I hate myself,” scored 0–2). Items are summed with a total score ranging from 0 to 56. For females, a raw score ≥ 20 is consistent with a T-score ≥ 65, which is a conservative recommended cut-off score for this measure. The CDI has strong psychometric properties and has been validated for use in children and adolescents with chronic illness [28]. Internal consistency in the current sample was very good (*α* ≥ .87).

The 21-item Beck Depression Inventory-II (BDI-II) was administered to participants 18 years and older to assess depressive symptoms over the previous two weeks [29]. Participants endorse one of four statements (e.g., “I do not feel sad,” “I feel sad,” “I am sad all the time and I can’t snap out of it,” “I am so sad and unhappy that I can’t stand it,” scores 0–3). Items are summed for a total score ranging from 0 to 63. A total score between 19 and 29 reflects moderate depression and a total score ≥ 30 reflects severe depression [30]. The BDI has been validated in adults with chronic illness [31]. Internal consistency in the current sample was very good (*α* ≥ .86).

##### Anxiety symptoms

The 41-item Screen for Child Anxiety Related Disorders (SCARED) self-report form was administered to assess anxiety symptoms over the previous three months [32]. Items are scored on a 3-point Likert scale (00 = *not true or hardly ever true*, 2 = *very true or often true*) and summed to create a total score ranging from 0 to 82. A total score ≥ 25 reflects clinically significant anxiety. Sample items include “I am a worrier” and “I worry about how well I do things.” The SCARED has been well validated for children ages 8–18 with chronic conditions. Although several participants were > 18 years, content level review by doctoral level psychologists suggested items were appropriate to administer to young adults. Internal consistency in the current sample was excellent (*α* ≥ .94).

#### Pain intensity

Participants rated their average pain intensity over the previous two weeks using a Numerical Rating Scale (NRS**)** with a range of values from *no pain* (0) to *worst pain imaginable* [10]. The NRS is a well-utilized measure of pain intensity in youth and adults [33, 34]. A pain rating ≥ 4 is used as a clinical cutoff value for the current study.

### Data analytic plan

The TEACH protocol was refined throughout the study using an iterative process, with changes made in response to patient feedback and interventionist observations. Protocol feasibility was assessed by attendance/completion rates and through themes that emerged from the qualitative interviews. Given the small sample size, non-parametric tests of difference (i.e., Wilcoxon signed-rank test) were conducted to test whether reductions in fatigue, psychological distress, and pain at T2 were statistically significant. For depressive symptoms and fatigue, analyses were only conducted for those under age 18 (*n* = 10) as the measures administered differed based on patient age (see Quantitative Measures section for additional details). Given that only four participants 18 years or older completed TEACH (see Results), statistical differences between T1 and T2 were not assessed for fatigue and depressive symptoms.

## Results

### Clinical characteristics

Means and standard deviations among study variables at baseline (*n* = 18) are presented in Table 1. In general, patients had moderate symptoms of fatigue, depression, anxiety, and pain.

**Table 1:** Time 1 means and standard deviations among study variables at baseline (*n* = 18)

	Obs Range	Possible Range	Clinical cut point	*M*	*SD*
Participants under 18 years, *n* = 10					
PROMIS Fatigue	2–34	0–40	≥ 26	20.00	10.46
Children’s Depression Inventory	5–24	0–56	≥ 20	17.44	7.75
SCARED	7–48	0–82	≥ 25	26.80	14.52
Pain Intensity	0–7	0–10	≥ 4	3.10	2.67
Participants ≥18 years, *n* = 8					
PROMIS Fatigue	20–39	8–40	≥ 32	29.50	13.44
Beck Depression Inventory	5–34	0–63	≥ 19	15.33	10.02
SCARED	5–52	0–82	≥ 25	27.00	23.64
Pain Intensity	2–7	0–10	≥ 4	3.33	1.15

*Obs* observed, *PROMIS* patient reported outcomes measurement information system, *SCARED* Screen for Child Anxiety Related Disorders

Disease severity based on the SLEDAI indicated minimal disease involvement (mea*n* = 4.33, SD = 4.42, range 0–13) at the time of enrollment for all participants.

### Recruitment and retention

Approximately 50% of participants agreed to participate. Eighteen of 34 (53%) consented and were enrolled in the intervention after meeting eligibility criteria. Of these, one withdrew prior to starting treatment due to scheduling difficulties and three withdrew after session 2. Thus, the total retention rate for individuals who started treatment was 82.4%. A total of 14 female participants (ages 13–19 years, 4 ≥ 18 years, *M* = 16.21, *SD* = 2.05) completed the 6-session protocol. Young adults with cSLE were invited to bring a caregiver or significant other to three treatment sessions. Among treatment completers, twelve participants (86%) brought a parent, one participant age 18 or older brought a significant other (i.e., boyfriend), and one participant over 18 years completed the intervention without direct support from a caregiver or significant other.

### Refinement of the TEACH protocol

#### Step 1: Deliver initial version TEACH and identify additional content areas

The initial version of TEACH was delivered to three participants. Following treatment delivery, based on patient feedback and interventionist recommendations, additional content addressing medication adherence and parent-child communication strategies was added. Minor modifications were made to expand upon sleep hygiene content. Further, problem-solving was revised to be more specific to concerns of youth with cSLE (e.g., difficulty getting out of bed).

#### Step 2: Deliver second version of TEACH and identify further areas of modification

The second version of TEACH was delivered to five participants. Following treatment delivery, additional content areas, including identifying and challenging automatic thoughts, and advocating for self, were either revised or added to the protocol. Mindfulness meditation was also added as a relaxation strategy (e.g., mindful breathing) and with the goal of enhancing the effectiveness of other strategies taught in the program (e.g., mindfully engaging in pleasant activities).

Given the current study included individuals with cSLE both over 18 and under 18, distinct protocols were developed during this phase to better address developmental differences between these groups. Specific modifications for young adults with cSLE included a greater emphasis on autonomy with symptom self-management and incorporating a significant other as opposed to a caregiver, in three of the six sessions. Moreover, interventionists flexibly delivered treatment content (e.g., greater emphasis on sleep hygiene vs. medication adherence for those who reported more sleep difficulties than problems with taking medication, as an example) based on the specific needs of each young adult participant.

#### Step 3: Deliver third version of TEACH

The final version of the TEACH protocol was administered to six remaining participants. Table 2 details a list of strategies delivered in the final version of TEACH.

**Table 2:** Modified cognitive behavioral protocol for adolescents and young adults with cSLE.

Session Number	Attendees	Adolescent Content	Young Adult Content
1	Participant and Caregiver^a^	Introduction and overview of program Psychoeducation Caregiver/significant other guidelines	
2	Participant	Activity pacing	
		Relaxation Sleep hygiene	Pleasant activity planning *Communication strategies*
3	Participant (plus caregiver for adolescents)	Pleasant activity planning Progressive muscle relaxation Caregiver check-in *Communication strategies*	Medication adherence Sleep hygiene Relaxation
4	Participant (plus Caregiver^a^ for young adults)	Mindfulness Identifying automatic thoughts Common thought traps	
			Caregiver/ significant other check-in
5	Participant	Challenging automatic thoughts Calming statements Problem solving	
6	Participant and Caregiver^a^	Advocating for self Maintenance plan	

*Notes.* One in-person, 60 min session per week administered by doctoral level psychologist

^a^Participants ≥18 years invited to bring significant other, roommate, close friend, or caregiver

Content in italics is optional

### Qualitative interviews

Qualitative feedback was obtained from all completing participants regarding program feasibility, acceptability, and patient outcomes. See Table 3 for representative quotes from participants during the qualitative interviews organized by domain and theme.

**Table 3:** Representative Quotes from Qualitative Interviews

**Domain 1: Feasibility**	
Theme: Practice/Usability of skills	
*I think [the skills] are pretty versatile*. *.*. *you can do them while you are in a room full of people and they might not necessarily know.* …*before I knew about activity pacing I would just try to squeeze it in as much as I possible could without breaking and then I’d just wear myself out and that would lead to flares. But I feel better now that I’m taking breaks in between.* *Out in the sun, I can’t go and do anything so when my friends are playing in the sun, so the [calming] statements come in handy.*	
Theme: Barriers to Feasibility	
*If I’m out with friend, I probably wouldn’t want to do [the coping skills] in front of them.*	
**Domain 2: Tolerability/Acceptability**	
Theme: Program Format	
*There wasn’t too much information packed in one session, but it also wasn’t so short that there was no time to go over anything.* *Personally, I liked six [sessions] because that still really kept me going and now, being the last session, I feel awesome. I feel like I can actually move on…* *There could probably be a couple more sessions just to go over certain things and go over things that you think you need a little more help on.*	
Theme: Therapist Characteristics	
*Like when she did [the coping skills] with me. I liked that because I felt like… me trying to do it at home probably wouldn’t have worked so I liked how she did it with me here just so I could know what it feels like.* *She focused more on what was specific for me. It wasn’t overall what was helpful for everybody- it was specific for me.* *I felt like [the therapist] actually understood where I was coming from which was amazing*. *.*. *it’s so nice to have someone to listen to that*	
Theme: Suggested Modifications	
*Post-it notes to write out things and a specific time and place to do a certain activity [would be helpful].* *…advocating for yourself should have been one of the first skills instead of the last skill because I think that’s something teens could use a little more practice on.* *[More skills for] memory [which] is like a big issue for me and it gets me a lot, it gets frustrating.*	
**Domain 3: Treatment Outcomes**	
Theme: Sleep/Fatigue	
*[Using the sleep skills] helps me take less medicine at nighttime. Instead of taking melatonin, I just used the relaxing muscles [progressive muscle relaxation].* *So when I tried [charging my phone outside of my room]…I tried it for a full week and it actually worked. And I liked it so I want to continue with that because I actually slept better.*	
Theme: Mood	
*[The intervention] gave good things to do to keep stress levels down and to better take care of myself in the future.* *Just like changing your perspective on things*. *.*. *she just said like if you have a negative thought then you can turn it into a positive like this and then she gave different ways.* *And I feel like I’m more happy, a lot happier than what I was before I started.*	
Theme: Pain	
*I think [the intervention] is really helpful with pain and how to deal with that.* *[Progressive muscle relaxation is helpful when] I have aches… or feel tight.* *I definitely think [the content from the intervention is] going to help me a lot, especially when I am having a flare.*	
Theme: Self-Management	
*[The program provided] good ideas to calm down and take care of myself- not just laying around and feeling bad, but preventing feeling bad in the future.*	
Theme: Self-Efficacy	
*I’m actually very confident that this program will help me cope better.* *…when I do feel sick I try to tell myself like oh everything’s going to be okay. You don’t have to be sad all the time. I actually really needed that.*	

#### Domain 1: Feasibility

Several feasibility themes emerged involving the practice/usability of skills and barriers to feasibility. Participants reported that the skills were generalizable and easy to integrate into their routine. Participants reported favoring deep breathing, activity pacing, and cognitive restructuring (including calming statements) most frequently of all skills taught in the program. Commonly reported barriers to TEACH interventions included forgetting to consistently practice the skills outside of sessions and reluctance to practice skills in front of peers.

#### Domain 2: Acceptability

Several themes pertaining to acceptability emerged during the qualitative interviews. These included program format, therapist characteristics, and suggested modifications. With regards to program format, participants generally enjoyed the pace of the program and most noted that the six-session format of the intervention was beneficial. Some participants suggested a longer protocol to enhance learning/application of skills. In terms of therapist characteristics, participants reported enjoying the therapist empathizing with their feelings, practicing skills alongside them, and tailoring the protocol to fit their needs. Suggested program modifications included the use of additional prompts/practice reminders, introducing self-advocacy earlier in the protocol, and including additional content to address participant memory problems.

#### Domain 3: Outcomes

The outcomes that emerged as most effectively managed due to the intervention included sleep difficulties/fatigue, mood symptoms, and pain. Participants reported the sleep hygiene skills in particular improved their overall fatigue and quality of sleep. Further, the skills taught in the intervention improved mood symptoms, stress management, and pain symptoms. In addition, participants reported increased self-management and self-efficacy. Specifically, participants reported use of self-management to address future difficulties and increased confidence in their ability to use the coping skills taught effectively.

### Initial effectiveness

Results are reported in Table 4. Significant reductions were found for fatigue and depressive symptoms among participants under 18 who completed the TEACH protocol. Descriptive data suggest that fatigue and depressive symptoms improved at T2 for participants 18 years or older though these data were not explicitly analyzed due to a small sample size. There was a trend towards reduction of pain levels from T1 to T2 (*p* = .056). Participants who completed TEACH also experienced a marginal reduction in anxiety symptoms at T2 (*p* = .051).

**Table 4:** Reduction in symptoms following TEACH protocol for intervention completers

		Mean T1	Mean T2	Average Decrease	Z	*p*	*r*
PROMIS Fatigue	≤ 17 years: *n* = 10	20.00	14.60	5.40	−2.81	.005	−.63
	≥ 18 years: *n* = 4	30.00	16.25	13.75	–	–	–
CDI BDI	≤ 17 years: *n* = 9	17.44	11.60	5.84	−2.69	.007	−.62
	≥ 18 years: *n* = 4	20.00	6.50	13.5	–	–	–
Pain Intensity	*n* = 14	3.39	2.64	.62	−1.91	.056	−.36
SCARED	*n* = 14	27.43	23.29	5.20	−1.95	.051	−.37

*Note. r* indicates effect size for non-parametric tests of difference and is interpreted consistent with correlation coefficients; 0.1 indicates a small effect size, 0.3 represents a medium effect size, and 0.5 indicates a large effect size. *PROMIS* patient reported outcomes measurement information system, *CDI* Children’s Depression Inventory, *BDI* Beck Depression Inventory; *SCARED* Screen for Child Anxiety Related Disorders

### Exploratory findings: Referral rates

After the administration of TEACH, 50% of patients requested to receive additional psychological care in a clinical setting to continue maintaining treatment gains and were successfully referred for such services. These participants had not previously been involved in the psychological management of symptoms associated with their cSLE.

## Discussion

Findings suggest that TEACH, a tailored CBT intervention for AYA with cSLE, is feasible and potentially effective. Over 80% of participants who started the intervention completed the program, which provides evidence of feasibility and acceptability. Participants and interventionists provided general feedback throughout the study which was used to help revise the protocol in an iterative manner. Furthermore, participants generally provided positive feedback on the feasibility, acceptability, and effectiveness of the TEACH protocol during qualitative interviews.

Treatment completers also evidenced significant reductions in fatigue and depressive symptoms. This is important given that fatigue and depressive symptoms commonly occur in individuals with cSLE [5, 6], and are associated with adverse outcomes in youth, such as diminished health-related quality of life [6]. Of note, reductions in pain and anxiety following TEACH did not reach levels of statistical significance but there were notable trends in symptom reduction. Overall, this pilot investigation suggests that TEACH may be both a practical and potentially beneficial in improving patient outcomes.

Throughout the study, modifications to the TEACH protocol were made based on participant feedback and interventionist observations. Following initial delivery of TEACH, we subsequently included content addressing medication adherence, parent-patient communication strategies, identifying and challenging automatic thoughts, and self-advocacy. Modifications were also made to better target the unique needs of young adults versus adolescents with cSLE. For example, in the young adult protocol, strategies to help participants self-manage their medication are explicitly discussed. In the adolescent protocol, medication management is discussed with the goal of gradually promoting independence with scaffolded support from caregivers. Related, adolescents versus young adults may differ in the type of primary support they receive. The majority of adolescents reside within their familial home. As such, they generally receive support from a parent or caregiver. Conversely, young adults with cSLE may attend college or reside outside of the familial home and therefore may identify primary support from a significant other such as a romantic partner, or may not identify an external support system at all. Thus, specifically incorporating the different types of support commonly experienced by an adolescent versus a young adult was essential. Finally, skills such as activity pacing and advocating for self were tailored to address the unique demands of young adulthood (e.g., balancing work and college courses) relative to adolescence.

Overall, results from qualitative interviews suggest that participants generally found the intervention to be feasible, acceptable, and effective, particularly for the management of sleep/fatigue, mood issues, and pain symptoms. In addition, participants reported increased self-management and improved self-efficacy when using the coping skills learned. However, it is noteworthy that only around 50% of patients approached ultimately participated in the study, which suggests that some patients would benefit from another treatment format that is less burdensome of patients and families, such as a telehealth format [35]. However, it may be critical for such a telehealth format to include interactive elements as the heavily web-based approach used in the only previously studied cognitive behavioral treatment of cSLE was unsuccessful in improving patient outcomes [12]. Further, approximately 50% qualified upon screening. Thus, relaxing some screening criteria (e.g., using a clinical cut-off score of 13 vs. 20 for the CDI [27]) in future research to increase the number of patients who would be eligible for treatment may be warranted.

Results provide preliminary support that TEACH is particularly effective in improving fatigue and mood symptoms in AYA diagnosed with cSLE. Thus, future randomized clinical trials designed to systematically test the effectiveness of TEACH may focus on fatigue and mood as primary outcomes. As the TEACH protocol failed to significantly reduce pain (similarly to the one previous CBT study for adolescent females [12]) and anxiety symptoms, further revision of the intervention to more directly target these symptoms may be warranted. Given that disease activity was low from the outset, the reductions in fatigue and depressive symptoms over the duration of the study were likely *independent* of disease activity.

Additionally, though not a primary outcome of the current study, we found that TEACH was a successful launching point to establish longer term mental health treatment for youth with cSLE. We successfully referred 50% of patients to receive psychological care after the completion of the intervention. None of these patients had previously been engaged in psychological treatment related to their cSLE symptoms, thus this may be a further indicator of treatment success. In terms of other clinical implications, it may be beneficial to offer screening and brief psychoeducation during an initial medical visit prior to triaging patients to such an intervention, which is an approach that our research team has successfully employed in the management of other pediatric health conditions that involve pain [36]. Youth with cSLE who screen positive on symptoms of fatigue and depression, which are explicit targets of the TEACH intervention, may yield an optimal benefit from this approach to care.

Given that our recruited sample is not entirely representative of the predominantly African American female population that are affected by SLE [37], it will be critical to enhance the cultural sensitivity of the intervention approach in future research. This may include engaging representative patients and families as co-investigators or research collaborators to further enhance the feasibility and effectiveness of the intervention. By inviting patients to participate in the refinement of the intervention approach as key stakeholders, in addition to applying learnings from intervention research of other chronic health conditions where minority populations are disproportionally affected (i.e., pediatric sickle cell disease) [38, 39], we may be able to further enhance patient recruitment, retention, engagement, and ultimately patient outcomes. A telehealth application of the protocol, for example, may increase program feasibility and reduce participant bias. Reaching a broader demographic of patients affected by cSLE (e.g., males, lower resourced patients) will also be critical to enhancing the feasibility and effectiveness of treatment. Future directions may also address the transition from pediatric to adult health care. Youth with cSLE and co-occurring mood symptoms may particularly benefit from collaborative transition planning well in advance of their anticipated transition to adult care.

## Conclusions

In conclusion, TEACH holds promise as a feasible and potentially effective approach to target the unique needs of AYA with cSLE. TEACH is now ready for additional refinement and testing in a controlled trial.

## Abbreviations

ACR: American College of Rheumatology
AYA: Adolescents and young adults
BDI-II: Beck Depression Inventory-II
CBT: Cognitive behavioral therapy
CDI2: Children’s Depression Inventory 2nd Edition
cSLE: Childhood-onset systemic lupus erythematosus
SLE: Systemic lupus erythematosus
NRS: Numerical Rating Scale
SCARED: Screen for child anxiety related disorders self-report form
TEACH: Treatment and Education Approach for Childhood-onset Lupus
